# Influence of Topography on the Site Selection of a Moon-Based Earth Observation Station

**DOI:** 10.3390/s21217198

**Published:** 2021-10-29

**Authors:** Guoqiang Chen, Huadong Guo, Yixing Ding, Haolu Shang, Mingyang Lv, Ke Zhang

**Affiliations:** Key Laboratory of Digital Earth Science, Aerospace Information Research Institute, Chinese Academy of Sciences, Beijing 100094, China; chengq@radi.ac.cn (G.C.); hdguo@radi.ac.cn (H.G.); shanghl@radi.ac.cn (H.S.); lvmy@aircas.ac.cn (M.L.); linealge@outlook.com (K.Z.)

**Keywords:** Moon-based observation station, Earth observation, sun light, fuzzy evaluation

## Abstract

The Moon provides a long-term, stable, and unique location for Earth observation. Several space agencies, such as NASA, ESA, and CNSA, have conducted lunar explorations. To build a Moon-based observation station, site selection is the first step. The time coverage of Earth observation, e.g., the whole Earth disc observation or Earth-related plasmasphere and magnetosphere, the duration of sunlight coverage, and topography (i.e., slope) are the three major factors influencing site selection, especially in the Moon’s south pole region. In this study, we used the Chang’E digital elevation model (DEM) together with Earth, Moon, and Sun positions deduced from JPL ephemeris for site selection. Two craters, Faustini and Shoemaker, were chosen for the fuzzy evaluation of these three factors based on a multiple-input single-output (MISO) model during a 19-year period. The results show that the edge regions of craters and small hills, potholes, or uplifts inside craters are unsuitable for a Moon-based observation station. The south pole area, including these two craters, has relatively low time coverage of sunlight and some unevenly distributed, permanent shadow areas. This indicates a low thermal environment for radiation protection, whereas the relatively flat topography and the ability to cover a field of view several times the Earth’s radius enable observations of the plasmasphere and magnetosphere.

## 1. Introduction

With humans’ growing appetite for lunar resources and booming lunar exploration activities, public interest in lunar research has again increased. Recently, several countries have announced their Moon exploration programs. For instance, the ESA has a program named Moon Village, which aims at establishing a Moon base for science, business, mining, and even tourism through international collaboration [1]. NASA has announced a lunar landing mission called Artemis, which is expected to be completed by 2024 [2]. China has also undertaken decades of Chang’E planning [3,4]. In these lunar exploration programs, Earth observation from a Moon site is one of the main tasks. Existing studies have revealed that Moon-based Earth observation provides longevity, integrity, and stability advantages over satellites in observing the solid Earth tide, global energy budget, climate and environmental change, and even the near-Earth space environment [5,6]. Furthermore, Moon-based Earth observation is unique in its ability to monitor phenomena related to Earth–Moon interaction [7,8]. Due to the advantages and uniqueness of Moon-based Earth observation, a broadband radiometer and an array spectrometer will be settled on the lunar surface to conduct a long-term Earth radiation budget experiment in this decade [9,10].

**Figure 1:**
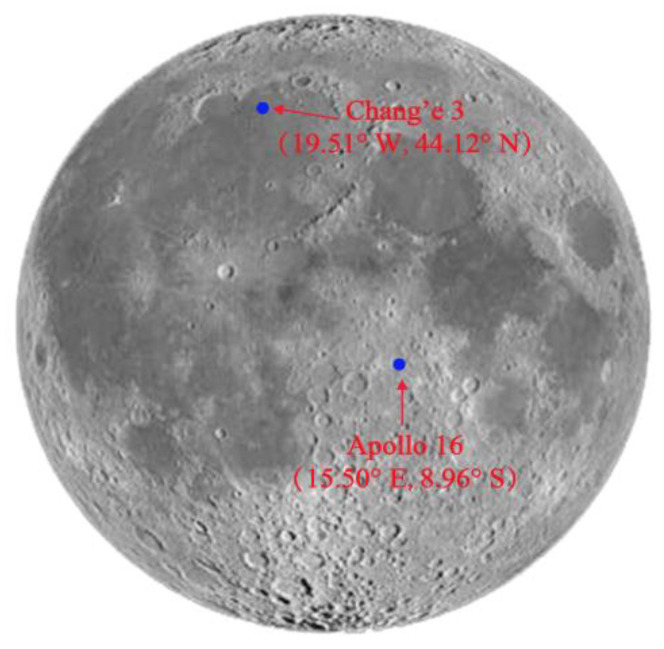
The landing sites of Apollo-16 and Chang’E-3.

Unlike space-borne Earth observation, Moon-based Earth observation is facing the problem of where to locate the equipment on the lunar surface. Because only a few short-term experiments have been conducted in the past, the suitability of establishing Earth observation stations at different sites on the Moon has not been fully discussed. In 1972, the Apollo 16 astronauts used a far-ultraviolet camera/spectrograph to obtain images of the Earth’s atmosphere and corona [11]. In 2013, the lander of the Chang’E-3 mission successfully brought an extreme ultraviolet (EUV) camera to the lunar surface and performed one-year Earth plasma observation [12,13]. The locations are labeled in Figure 1.

In Moon-based Earth observation, three factors play decisive roles in site selection due to the topography of the Moon’s surface. The first one is the visibility of the whole Earth disc. Whether the mission objective is to obtain an image of the Earth or to measure the Earth’s outgoing energy, the sensor must be able to observe the whole Earth for as long as possible. The literature shows that most places on the nearside of the Moon have 100% temporal coverage of the whole Earth disc except the edge areas [14]. The visibility of the lunar polar area from Earth, especially the lunar south pole area, has not yet been investigated in detail. The lunar south pole area is one of the hottest candidate sites for a permanent Moon base [15,16]. A base inside one crater at a high lunar latitude would have wide Earth disc observation coverage, and the sun would always be low on the horizon; this situation benefits Earth photometric observation [17]. Another factor is the sunlight. Sunlight brings energy to the sensors, but also introduces thermal noise. Particularly, for radiation measurement and radar observation, thermal noise is the key factor affecting the quality of observation data. Therefore, long-term shadowed areas in the lunar south pole may be more suitable for an observatory. The third factor is the surface slope. Earth observation equipment cannot be placed where the slope is too steep.

From the research on Moon topography, we know that the Moon is not a smooth sphere, but has a complex surface composed of many craters [18,19]. Research attention has mainly focused on the relationship between topography and the distribution of resources such as ice [20]. Among the various engineering practices, Moon topography is an important factor that should be considered. For example, site topographic mapping and rover localization, the subsurface structure, and the stratigraphy of the landing site were discussed for the Chang’E probe [21,22,23]; topography data were also considered for other landing detectors, such as Apollo and Lunar [24,25,26]. In a Moon site selection task focusing on Earth observation, complex topography may occlude sight and sunlight [27]; therefore, Moon topography cannot be ignored during site selection.

Considering Moon topography, we propose a site selection method for a Moon-based station. The whole Earth disc and Earth-related plasmasphere/magnetosphere are both observation objects of interest for a Moon-based station. In this paper, the whole Earth disc as observation object is used to explain the evaluation model; three main factors, including the surface slope, the sunlight, and the visibility of the Earth disc, are integrated in our analysis. To comprehensively consider the above factors, a multi-factor fuzzy evaluation (MFE) method based on a multiple-input single-output (MISO) controller is proposed. This controller inputs the membership of multiple factors, and outputs the possibility of selection.

This paper is organized as follows: Section 2 provides a detailed analysis of the Moon’s topography, the time coverage of sunlight, and the visibility of the whole Earth disc from a Moon station, and provides a detailed introduction of the MISO model. Section 3 illustrates the influence of topography on site selection in the south pole area, and describes a detailed application of the evaluation model introduced in Section 2. Because the nutation period caused by the Moon and the cycle of the lunar orbit’s ascending node is about 18.6 years, to analyze the influence of topography, we used a simulation period of 1 January 2001 to 31 December 2019. The last section outlines our conclusions based on Section 3.

## 2. Site Selection Method

**Figure 2:**
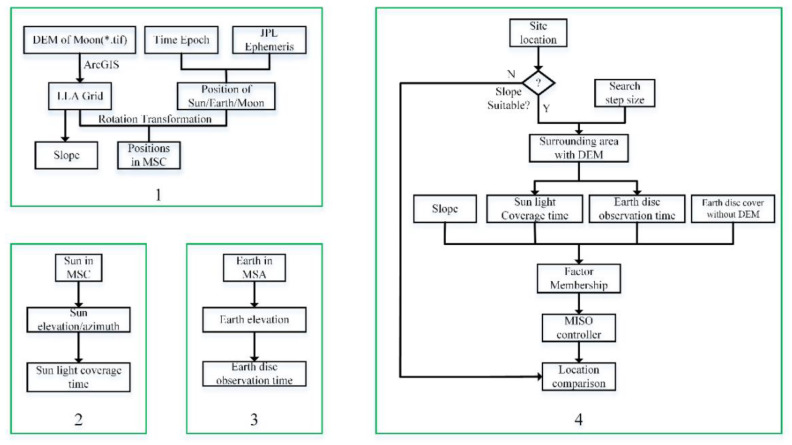
The flow chart of this study.

To choose a site suitable for Earth observation on the Moon’s surface, detailed data on Moon surface slope, the time coverage of sunlight, and the time coverage of Earth disc observation were considered. Figure 2 depicts the flow chart of deriving these three factors from JPL ephemeris and the Chang’E-2 DEM [28]. The DEM’s resolution is 50 m, and was obtained from a CCD stereo camera at an orbital height of 100 km from October 2010 to December 2010. Box 1 shows the data processing of the lunar topography file and JPL ephemeris. Then, we obtained the area’s latitude, longitude, altitude, slope, and the positions of the research objects in Moon Station Center (MSC) coordinates. A detailed MSC definition is provided in Section 2.2. Box 2 shows the process of obtaining the sunlight coverage. Box 3 shows the process of obtaining the Earth disc observation time coverage in Moon Station Antenna (MSA) coordinates. Box 4 shows the process of site selection through a MISO controller; the small box labeled “Earth disc cover without DEM” calculates the disc coverage threshold, which helps to define the Earth disc observation time. A detailed MISO definition is provided in Section 2.4. In this paper, the coordinate system names (e.g., MSC and MSA) used in variables, vectors, or formulas are represented by lowercase letters to ensure a uniform and standardized display, and vectors are shown in black bold type.

### 2.1. Moon Surface Slope

The existing DEM of the Moon surveyed by Chang’E sensors shows many craters with different topographies are spread all over the Moon’s surface. For a Moon-based Earth observation station, a relatively gentle slope is better for construction convenience and disaster avoidance. The surface slope can be derived from the gradient of each cell of the Moon’s DEM grid.

**Figure 3:**
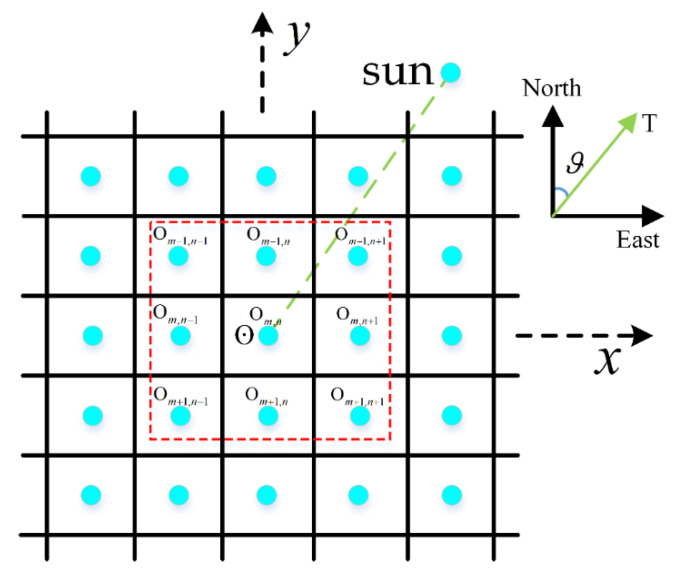
The DEM grid and MSC of a small area.

To calculate the gradient of surface at point $O_{m,n}$ in Figure 3, the DEM of $O_{m,n}$ and eight surrounding points are also extracted. Each surface point has slope $S_{w-e}$ in the west to east direction, and $S_{s-n}$ in the south to north direction. These are calculated as Equations (1) and (2), respectively:

(1) $$S_{w-e}=\frac{\left(O_{m+1,n-1}+2O_{m,n-1}+O_{m-1,n-1}\right)-\left(O_{m+1,n+1}+2O_{m,n+1}+O_{m-1,n+1}\right)}{8\cdot dx}$$

(2) $$S_{s-n}=\frac{\left(O_{m+1,n+1}+2O_{m+1,n}+O_{m+1,n-1}\right)-\left(O_{m-1,n+1}+2O_{m-1,n}+O_{m-1,n-1}\right)}{8\cdot dx}$$

where dx and dy are the resolution of every grid point on the *x*-axis and *y*-axis, respectively; subscripts m and n are the row number and column number, respectively. For a suitable Moon station site, the slope should subject to the following constraint:

(3) $$\left|S_{w-e}\right|<S_{max},\;S_{s-n}<S_{max}$$

where $S_{max}$ is the maximum slope value, set in advance. This maximum slope is derived according to the construction feasibility of the surface conditions on Earth, which are detailed in Section 2.4.

### 2.2. Sunlight Time Coverage

A relatively simple method for calculating the time coverage of sunlight is proposed. A coordinate of Moon Station Center (MSC) is framed on the assumed center station point O, the *x*-axis is parallel to the latitude line of the Moon and directed to the east, the *y*-axis is parallel to the longitude line of Moon and directed to the north, and the *z*-axis obeys the right-hand rule together with the x- and y-axes. Here, the positions of the Moon center M, the Sun center S, and the Earth center E are derived in MSC, and the corresponding positions in MSC are $M^{msc}$, $S^{msc}$, and $E^{msc}$, respectively. The bore-sight of sunlight from center point *O* is $S^{msc}$, and its vector components are $S_{x}^{msc}$, $S_{y}^{msc}$ and $S_{z}^{msc}$.

For any Sun position, the sun elevation angle ϕ and azimuth angle ϑ can be expressed, respectively, as:

(4) $$\phi \;=\cos{\left(\frac{S_{z}^{msc}}{\left\|S^{msc}\right\|}\right)}^{-1}$$

(5) $$\phi =\{\begin{array}{c} \cos{\left(\frac{S_{y}^{msc}}{\left\|P\right\|}\right)}^{-1},\;\cos{\left(\frac{S_{x}^{msc}}{\left\|P\right\|}\right)}^{-1}\leq \frac{\pi }{2}\\ 2\pi -\cos{\left(\frac{S_{y}^{msc}}{\left\|P\right\|}\right)}^{-1},\;\cos{\left(\frac{S_{x}^{msc}}{\left\|P\right\|}\right)}^{-1}>\frac{\pi }{2} \end{array}$$

where the symbol ‖‖ indicates the 2-norm of the variables, and P  is the projection of the $S^{msc}$ on the tangent plane, which can be expressed as:

(6) $$\;P=\left[S_{x}^{msc},{\;S}_{y}^{msc},\;0\right]$$

For a DEM grid, the azimuth angle of the surrounding point k to the center point O in MSC is expressed as $\vartheta_{k}$ in Equation (7).

(7) $$\vartheta_{k}=\{\begin{array}{c} \cos{\left(\frac{J_{y}^{k}}{\left\|J^{k}\right\|}\right)}^{-1},\;\cos{\left(\frac{J_{x}^{k}}{\left\|J^{k}\right\|}\right)}^{-1}\leq \frac{\pi }{2}\\ 2\pi -\cos{\left(\frac{J_{y}^{k}}{\left\|J^{k}\right\|}\right)}^{-1},\;\cos{\left(\frac{J_{y}^{k}}{\left\|J^{k}\right\|}\right)}^{-1}>\frac{\pi }{2} \end{array}$$

(8) $$J^{k}=\left[\Delta J_{x}^{k}\cdot dx,\;\Delta J_{y}^{k}\cdot dy,\;0\right]$$

where k is the surrounding point number; $J^{k}$ is a vector from center point O to surrounding point k; $J_{x}^{k}$ and $J_{y}^{k}$ are the components of $J^{k}$; $\Delta J_{x}^{k}$ and $\Delta J_{y}^{k}$ are the latitude difference and longitude difference, respectively; and dx and dy are the resolution of every grid on the *x*-axis and *y*-axis shown in Section 2.1, respectively.

The positions of surrounding points k in MSC can be expressed as $T_{k}^{msc}$ in

(9) $$T_{k}^{msc\;}=\left[\Delta J_{x}^{k}\cdot dx,\;\Delta J_{y}^{k}\cdot dy,\;D_{k}\right]$$

where $D_{k}$ is the DEM difference in the surrounding point k to center point O.

The Moon station will not be exposed to sunlight if Equation (10) is satisfied.

(10) $$\tan\left(\frac{D_{k}}{\left\|J^{k}\right\|}\right)>\phi$$

If more accurate altitude is needed, an interpolation algorithm, such as linear interpolation, might be introduced for the calculation of $D_{k}$.

### 2.3. The Visibility of the Whole Earth Disc from a Moon Station

**Figure 4:**
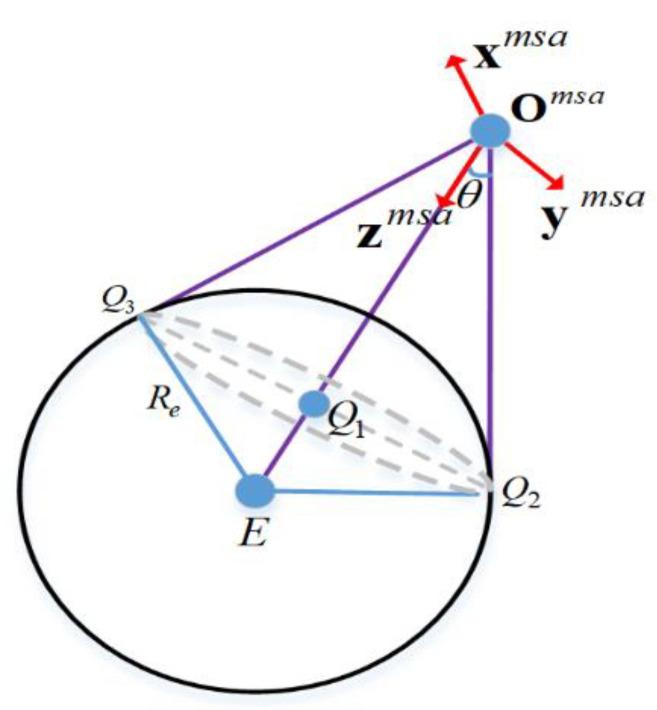
Observation of Earth disc from a Moon station.

An algorithm for determining whether the entire Earth disc can be observed is proposed here. Figure 4 shows a Moon Station Antenna (MSA) coordinate. $x^{msa}$, $y^{msa}$, and $z^{msa}$ are the three unit vectors perpendicular to each other in MSA; they follow the right-hand rule, and were transformed from MSC. $O^{msa}$ is the position of the Moon station in MSA, $z^{msa}$ is the optical axis directed towards the Earth’s center E. The margin of observation sight is a tangent to the Earth’s surface if there is no topographical occlusion of the Moon station. All the points of tangency form a secant plane, as indicated by the dotted ellipse in Figure 4. $Q_{1}$ is the intersection of $z^{msa}$ on the secant plane; $Q_{2}$ and $Q_{3}$ are two other tangent points; $Q_{1}$, $Q_{2}$ and $Q_{3}$ are three collinear points, as illustrated in Figure 4; and θ is the angle between surface tangent points and $z^{msa}$, which is calculated by Equation (11).

(11) $$\theta \;=\sin{\left(\frac{R_{e}}{\left\|E^{msa}\right\|}\right)}^{-1}$$

where $R_{e}$ is the average radius of the Earth and $E^{msa}$ is the position of the Earth’s center in MSA.

So, the positions of the surrounding points in MSA can be expressed as:

(12) $$T_{k}^{msa\;}{=\Xi }_{msc}^{msa}\cdot T_{k}^{msc\;}$$

where $\Xi_{msc}^{msa}$ is the transformation matrix from MSC to MSA. MSC and MSA have an identical center point O; $x^{msa}$ is perpendicular to the plane formed by $z^{msa}$ and $z^{msc}$. MSA is rotated from MSC by first rotating MSC around the *z*-axis with angle $\varpi_{1}$, and then rotating around the *x*-axis with angle $\varpi_{2}$, so the transformation matrix can be expressed by Equation (13).

(13) $$\begin{array}{l} \Xi_{msc}^{msa}=rotRx\left(\varpi_{2}\right)\cdot rotRx\left(\varpi_{1}\right)\\ \;=\left[\begin{array}{ccc} 1 & 0 & 0\\ 0 & \cos\varpi_{2} & \sin\varpi_{2}\\ 0 & -\sin\varpi_{2} & \cos\varpi_{2} \end{array}\right]\cdot \left[\begin{array}{ccc} \cos\varpi_{1} & \sin\varpi_{1} & 0\\ -\sin\varpi_{1} & \cos\varpi_{1} & 0\\ 0 & 0 & 1 \end{array}\right] \end{array}$$

The Earth disc will be sheltered if Equation (14) is satisfied.

(14) $$\frac{\pi }{2}-\tan{\left(\frac{T_{k}^{msa\;}\left(3\right)}{\sqrt{\|T_{k}^{msa\;}{\left(1)\right\|}^{2}+\|T_{k}^{msa\;}{\left(2)\right\|}^{2}}}\right)}^{-1}<\theta$$

If the observation object of interest is the Earth-related plasmasphere/magnetosphere, the observation area will be several (3~6) times the Earth’s radius, as shown in Figure 16. Then, the variable θ in Figure 4 should increase the same number of times for the calculation of time coverage.

### 2.4. The Evaluation Model

In this paper, a comprehensive multifactor fuzzy evaluation model is proposed for site selection [29]. The factor set is $U=\{u_{1},u_{2},\ldots u_{n}\}$; the subscript is the factor index.

Below, factor $u_{1}$ indicates surface slope $S_{i}$, and its fuzzy membership is $y_{1i}$. Factor $u_{2}$ indicates the sunlight time coverage $t_{s}$; its fuzzy membership is $y_{2i}$. Factor $u_{3}$ indicates Earth disc observation time $t_{e}$; its fuzzy membership is $y_{3i}$. Subscript i is the number of DEM grids shown in Figure 3; the DEM grid was obtained from the DEM data of Chang’E-2. Parameters $t_{s}$ and $t_{e}$ in Equations (16) and (17) can be calculated using a time series operation.

As for the Moon surface, the flatter the topography, the better the site. A site at which the slope exceeds a threshold $S_{max}$ is not a suitable location. Then, the fuzzy membership function for $u_{1}$ is expressed as:

(15) $$y_{1i}=\{\begin{array}{c} \frac{S_{max}-\left|S_{i}\right|}{S_{max}},\;0\leq \left|S_{i}\right|<S_{max}\\ \;0,\;\left|S_{i}\right|\geq S_{max} \end{array}$$

In the field of urban construction, a suitable slope for a residential building is around 0.3% to 10% [30]. Converted to degrees, a suitable slope is often below 5.71°, so the $S_{max}$ is set here to 5.71°.

For the factor $u_{2}$, a fuzzy membership function is expressed as:

(16) $$y_{2i}=\{\begin{array}{c} \frac{1}{\left(b-a\right)\cdot T}\cdot \left(t_{s}-a\cdot T\right),\;b\cdot T<t_{s}\leq a\cdot T\\ \frac{1}{b\cdot T}\cdot t_{s},\;0<t_{s}\leq b\cdot T\\ 0,\;t_{s}=0\;or\;t_{s}>a\cdot T \end{array}$$

where T is the total time window, a(0≤a≤1) is a scale coefficient corresponding to T, and b(0≤b≤a) is a scale coefficient indicating the most suitable time. The current highest efficiency of a large mono-crystalline silicon cell is 0.26; the efficiency of other common poly-silicon cells does not exceed 0.19 [31], so the coefficient b is set to 0.1 according to material photoelectric conversion efficiency and the time coverage of sunlight in craters, as analyzed in Section 3.2. Because the sunlight duration should be short for easier access to water and radiation protection, but it cannot be zero in order to enable access to solar energy, the coefficients a and b can be set to a paired change relationship, e.g., the coefficient a can be twice the value of coefficient b.

For factor $u_{3}$, the fuzzy membership function is expressed as:

(17) $$y_{3i}=\{\begin{array}{c} \frac{t_{e}}{\beta \cdot T},\;0\leq t_{e}\leq \beta \cdot T\\ \;0,\;t_{e}>\beta \cdot T \end{array}$$

where β is the scale coefficient and β⋅T is equal to the Earth disc observation duration without the DEM.

For each factor, four categories indicate Moon station suitability: high accessibility (HA), intermediate accessibility (IA), low accessibility (LA), and inaccessibility (IC). Each criterion is assumed to be of the Gaussian type.

**Figure 5:**
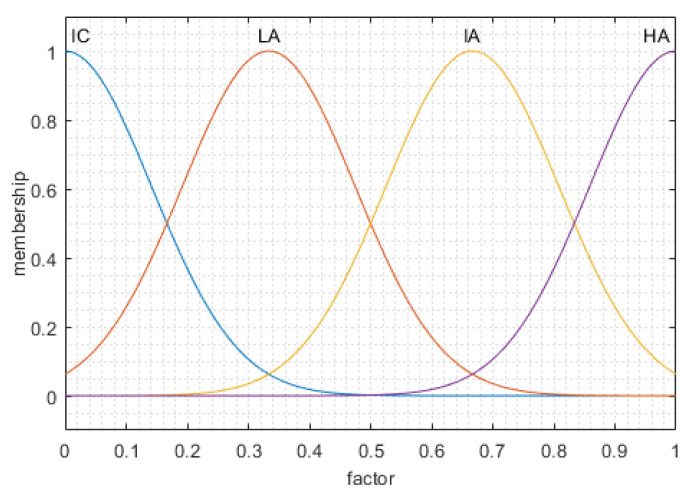
The relation diagram between the three factors and their membership.

**Figure 6:**
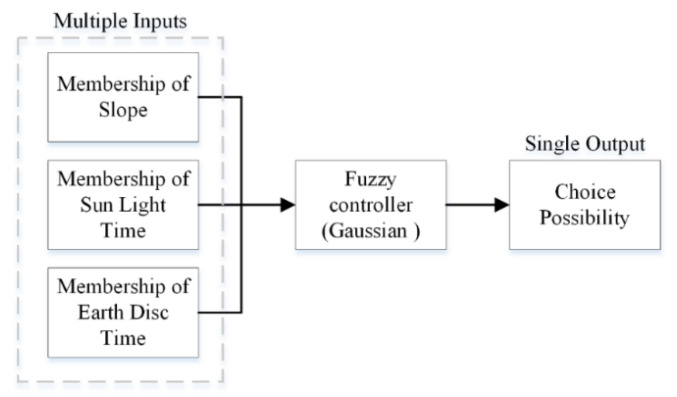
A diagram of the MISO fuzzy controller.

For factor $u_{1}$, the four categories are HA_1_, IA_1_, LA_1_, and IC_1_; for factor $u_{2}$, the four categories are HA_2_, IA_2_, LA_2_, and IC_2_. For factor $u_{3}$, the four categories are HA_3_, IA_3_, LA_3_, and IC_3_. The relationships of each factor and its membership satisfy the changes, as shown in Figure 5. The statement of MFE is “if $u_{1}$ and $u_{2}$ and $u_{3}$, then F”; the relation matrix is $R={\left(u_{1}\times u_{2}\times u_{3}\right)}^{T}\times F$, where T is the transpose matrix of the column vector and × indicates a Cartesian product. Through the MFE statement, a MISO diagram was constructed, as displayed in Figure 6. Multiple inputs can be converted to a single output through the controller.

For a Moon site, if $y_{1i}=0$, $y_{2i}=0$ or $y_{3i}=0$, the comprehensive evaluation index F is zero. For other sites, the comprehensive evaluation index F is calculated from the fuzzy rule tables shown in Appendix A.

In this paper, the factors $u_{1}$, $u_{2}$, and $u_{3}$ each contain four categories, so the fuzzy rule tables contain 64 rules. The fuzzy rule tables are shown in Appendix A.

## 3. Results and Discussion

### 3.1. The Influence of Topography on the Site Selection of a Moon-Based Observation Station

**Figure 7:**
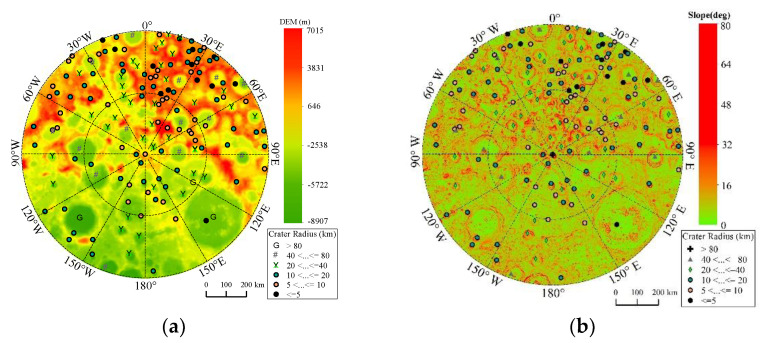
The surface topography distribution of the Moon from 70° to 90° S. (**a**) DEM distribution, (**b**) slope distribution.

The higher-latitude areas, especially in the lunar south pole region, are hot spots for a Moon station, ranging from 70° to 90° S. The DEM, the crater, and the slope distribution are shown in Figure 7. The original pixel scale of DEM is about 50 m in Figure 7. From Figure 7, we can observe that the topography on the front side is much more complex than the back side, since more craters are distributed on the front side, where the maximum slope is about 80.21° at very few sites; most of slope is lower than 40°. The DEM of higher-latitude areas is generally higher for the front side because of the uneven distribution of craters and the early interaction between the Moon and Earth [32]. For Earth disc observation in the south pole region, the Moon base should be established on the side facing the Earth, e.g., regions whose longitude mainly ranges from 90° W to 90° E. In subsequent sections, the DEM is considered because of the complex topography.

**Figure 8:**
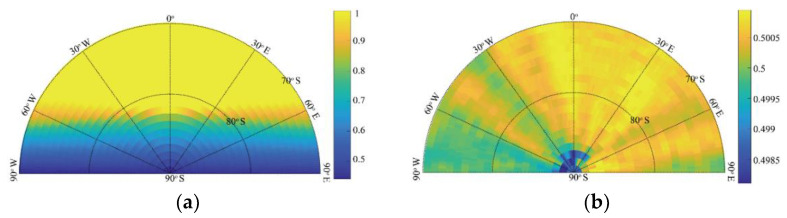
Time coverage of Earth disc observation (**a**) and sunlight (**b**) from 70° to 90° S for the nearside of the Moon surface without considering the DEM.

To study the influence of topography on the sunlight coverage duration and the visibility of the whole Earth disc, we first removed the influence of the Moon’s DEM, that is, the Moon was deemed a smooth sphere whose radius is about 1738 km. Figure 8 shows the time coverages of Earth disc observation and sunlight from 1 January 2001 to 31 December 2019. Since Earth observation research focuses on the front side area, the longitude range displayed in Figure 8 is only from 90° W to 90° E.

As shown in Figure 8a, areas at lower latitudes provide better Earth disc observation than higher-latitude areas; the Earth disc observation coverage drops sharply from almost 100% to lower than 50% when the latitude is greater than 80° because of the complex tide lock relation and the curvature occlusion due to the Moon’s surface. In Figure 8b, the duration of sunlight coverage is about 50% in higher-latitude areas, and varies little; the phenomena of polar day and night occur in south pole areas mainly because of the 1.54° difference between the Moon’s rotation axis and its revolution orbit. Figure 8 shows the time coverage of sunlight and Earth disc observation without considering the DEM. Note that craters are distributed on the Moon, as shown in Figure 7, so the DEM cannot be ignored in actual operation.

**Figure 9:**
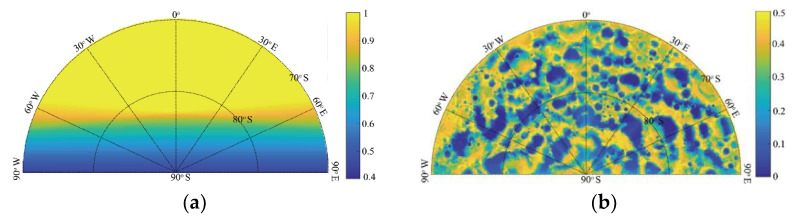
Time coverage of Earth disc observation (**a**) and sunlight (**b**) from 70° to 90° S for the nearside of the Moon’s surface considering the DEM.

We added DEM ranges from 70° to 90° S and from 90° W to 90° E to analyze the time coverage of sunlight and Earth disc observation. Because the resolution of the original DEM data file was about 50 m, we aggregated the original DEM into 4000 × 4000 m to reduce the amount of calculation (Figure 9). Then, the two distributions are shown in Figure 9. Because of the nutation period caused by the Moon, and given that the cycle of the lunar orbit’s ascending node is about 18.6 years, we used a simulation period of 1 January 2001 to 31 December 2019 to analyze the long-term influence of topography.

Figure 8 and Figure 9 show the polar projection. Because of the irregularities in the DEM, and the angle between the Moon’s rotation axis and its revolution orbit, a small change in altitude may significantly alter the lighting conditions on the Moon’s surface. The Moon’s surface topography shelters different areas from sunlight as the Sun’s elevation changes. This may produce permanently shadowed areas, such as the bottom of a crater, as shown in Figure 9b. This further illustrates the necessity of integrating the DEM into the analysis of sunlight duration for site selection.

The duration of Earth disc observation is less affected by DEM and its distribution is similar to the situation without the DEM. There are several reasons for this finding. Firstly, the field of view of the whole Earth disc between the Earth and Moon is only about 1.8°. This leads to a small field of view of the whole Earth in the sky. Secondly, most of the slopes are not very big, as shown in Figure 7, which leads to few sites being affected by occlusion. Third, the Moon is held by Earth’s tides, and only one side faces the Earth, which leads to a basically fixed line of sight for Earth observation. Fourthly, the reduction in resolution may decrease the difference because the amount of raw data for the range of 70° to 90° S of the nearside of the Moon is quite big, so coarse-resolution data were used to roughly analyze the differences in the time coverages of sunlight and Earth disc observation in large areas. When studying a specific area, such as a crater, coarse resolution data were not suitable, so the resolution used in Section 3.2 is 150 m.

Through the analysis in Section 3.1, we found that the distributions of sunlight, Earth disc observation, and topography differ on the Moon’s surface, so we chose several craters assessed previously for detailed analysis. The selected craters and other detailed analyses are described in Section 3.2.

### 3.2. Application of the Evaluation Model for Earth Disc Observation Site Selection

**Figure 10:**
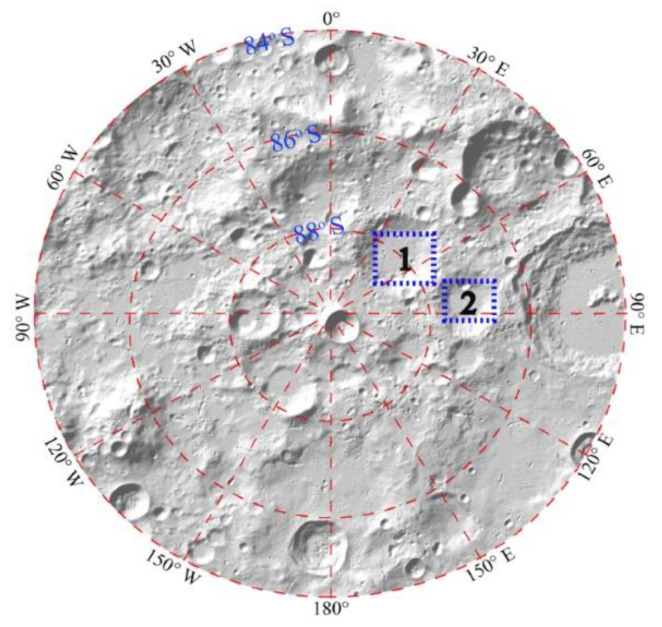
The distribution of the craters Shoemaker (in blue dotted frame 1) and Faustini (in blue dotted frame 2) on the Moon’s surface.

As the sun is relatively low on the horizon and the coverage of the Earth disc is not small when the Moon station is located in craters at high lunar latitudes, permanent shadowed regions may exist in these craters, and the sunlight coverage may be effectively reduced (but not to zero) in certain areas in these craters. So, in this study, two craters at high lunar latitudes were selected for analysis. The first crater, Faustini, is located at 87.3° S and 77° E, in one of the coldest areas, and it has higher LOLA, normal albedo, and is suspected to contain water [33]. The second crater, named Shoemaker, is located at 88.1° S and 44.9° E; the crater was hit by a lunar surveyor in July 1999, and the remote sensing detection results of microwave brightness temperature and the bolometric brightness temperature prove the presence of water ice deposits around the shadowed area [34]. From the results of the previous research, we analyzed potential sites in the Faustini and Shoemaker craters with the method described in Section 2.4. The locations of the two craters on the Moon surface are shown in Figure 10.

**Figure 11:**
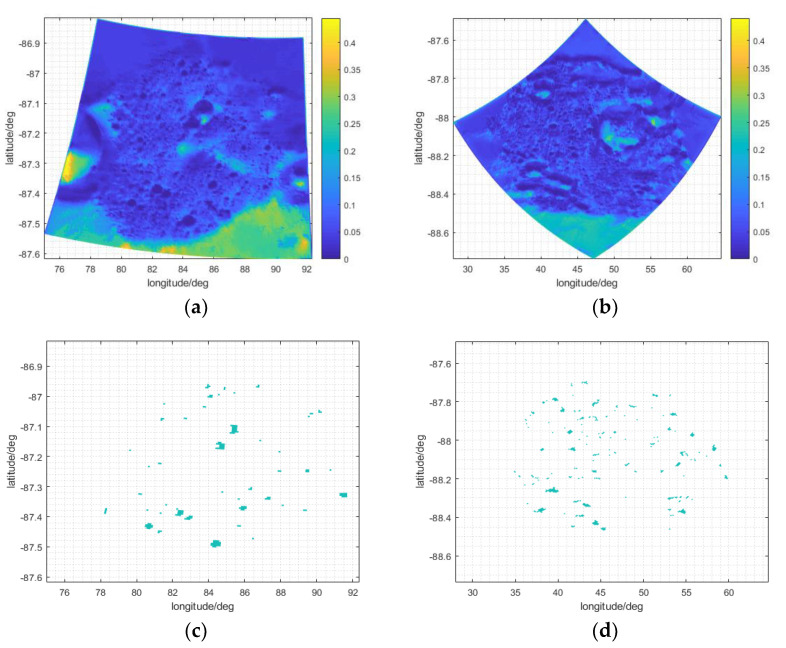
The time coverage of sunlight in (**a**) Faustini and (**b**) Shoemaker, and the permanent shadowed region (in irregular blue area) for (**c**) Faustini and (**d**) Shoemaker.

Figure 10 shows that the Faustini and Shoemaker craters are close to the south pole of the Moon, and are basically located in its eastern hemisphere. The radius and apparent depth of the two craters are moderate [35]: about 25.45 km and 2.36 km for Shoemaker and about 19.5 km and 2.17 km for Faustini, respectively. In the periodic motion of the Moon around the Earth, the Sun is relatively low on the horizon, and the polar day and night occur in the south pole area; the time coverage of sunlight and permanent shadowed regions is shown in Figure 11a–d. For Faustini, the permanent shadowed region accounts for about 4.21% of the total area, and for Shoemaker, the percentage is about 7.06%. The permanent shadowed region of Shoemaker is bigger than that of Faustini, mainly because Shoemaker’s DEM is smaller than Faustini’s, as shown in Figure 13a,b.

As shown in Figure 11a,b, the time coverage of sunlight in Faustini and Shoemaker is generally low and does not exceed 0.5. Because of the uneven topography in the two craters, as shown in Figure 13c,d, the distribution of sunlight coverage in the two craters is correlated with topography. For most of the Moon’s craters, the ring structure is caused by early meteorite impacts; basaltic lava flows are usually located in large impact basins [36], and the slope of the crater edge is usually higher than the inside area. Numerous small hills, uplifts, and potholes also appear at the bottoms of larger craters [37], which lead to steeper slopes in some places. In Figure 13c,d, the maximum slope is about 30°, which is only identified at a few sites. The slopes of the craters’ bottoms are generally lower than 5°, except for some small hills, potholes or uplifts, which occlude sunlight and reduce the time coverage of sunlight in the surrounding sites. For these surrounding sites, their azimuth is equal to the Sun’s, and their elevation is greater than the Sun’s, so some permanently shadowed regions exist at the bottom of Faustini and Shoemaker, as shown in Figure 11c,d. The Earth observation equipment will be protected from external noise and radiation in permanently shadowed regions. However, in terms of the energy cost for the equipment to work, permanently shadowed regions might not suitable for a Moon-based observation station. As presented in Equation (16), coefficient b is set to 0.1 according to the material photoelectric conversion efficiency and the time coverage of sunlight in craters.

**Figure 12:**
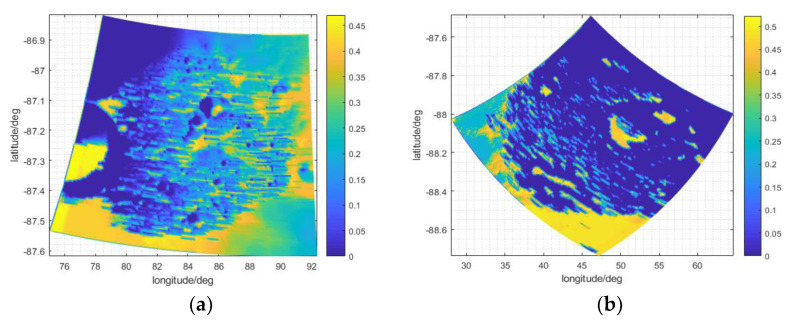
The time coverage of Earth disc observation in (**a**) Faustini and (**b**) Shoemaker.

**Figure 13:**
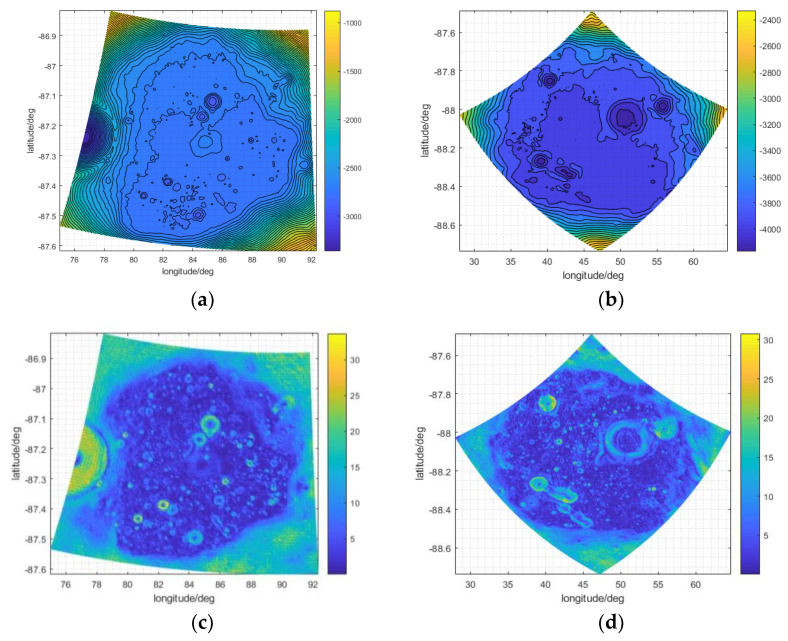
The distribution of the DEM in (**a**) Faustini and (**b**) Shoemaker, and overall distribution of slope in (**c**) Faustini and (**d**) Shoemaker.

Regarding the duration of Earth disc observation coverage, as shown in Figure 12, the uneven topography results in the uneven distribution of the time coverage of Earth disc observation at the bottoms of the craters. Because the two craters are located at high latitudes, and the relative elevation of the Earth is lower in these than in craters at low latitudes, small changes in topography more easily obstruct the line of sight to the Earth, and reduce the duration of Earth disc observation. Because the overall DEM of Shoemaker is lower than that of Faustini, as shown in Figure 13a,b, topography has more influence in Shoemaker than in Faustini. Because the Moon is held by the Earth’s tide, the azimuth of the Earth for a specific crater changes little, and its half field view of the Earth’s disc is about 0.9°; the time coverage of Earth disc observation changes little between the time periods.

Overall, the time coverage of Earth disc observation at high latitudes is generally less than 0.5, as shown in Figure 9a, but because the DEM resolution in Figure 9a is 4000 m × 4000 m, the finer details of the Earth disc observation duration are missed. When high-resolution data (150 × 150 m) are used (Figure 12), more details are provided.

The distributions of the time coverage of sunlight and Earth disc observation in Faustini and Shoemaker, together with hotspots’ properties, indicate that a suitable site could likely be identified in one of the two craters.

**Figure 14:**
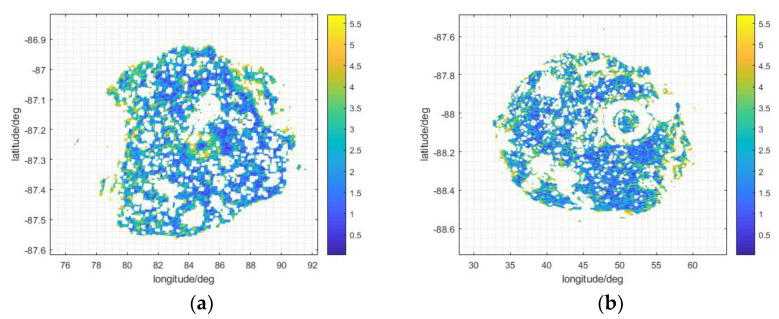
The distribution of slope within the set constraint in Faustini (**a**) and Shoemaker (**b**).

By applying the site selection method in Section 2.1, we found that the slope constraint influences its membership. According to the city building construction criterion in [30], a suitable slope should be below 5.71°; here, a suitable slope for the Moon station can also be assumed as being below 5.71°. The distribution of slope within that constraint is shown in Figure 14.

For most Moon craters, the slope of the crater edge inside small hills, uplifts, and potholes is usually steeper than other areas inside the crater. So, comparing Figure 13c with Figure 14a, and Figure 13d with Figure 14b, we can see that the crater edge areas and the insides of hills, uplifts, and potholes are mostly removed. The remaining regions are fragmented at the bottoms of the craters.

For Faustini, 42.6% of the total area has not been excluded, and for Shoemaker, the percentage is 23.15%. The radii of Shoemaker and Faustina are about 25.45 km and about 19.5 km, respectively; Shoemaker has a larger area than Faustini, so comparing the remaining areas in the two craters, Faustini is better than Shoemaker in terms of site selection. Because Shoemaker’s latitude is a little higher than Faustini’s, and the DEM of Shoemaker is lower than Faustini’s, the occlusion of the Earth disc bore-sight in Shoemaker’s edge area may be more serious than that of Faustini, preventing the observation of the whole Earth disc in edge areas for long periods of time. Though Faustini is closer to 90° E longitude than Shoemaker, which is the dividing line between the front and back sides of the Moon since the Moon is held by Earth’s tides, the Moon libration in latitudinal and longitudinal orientation still affects Earth disc observation capability.

**Figure 15:**
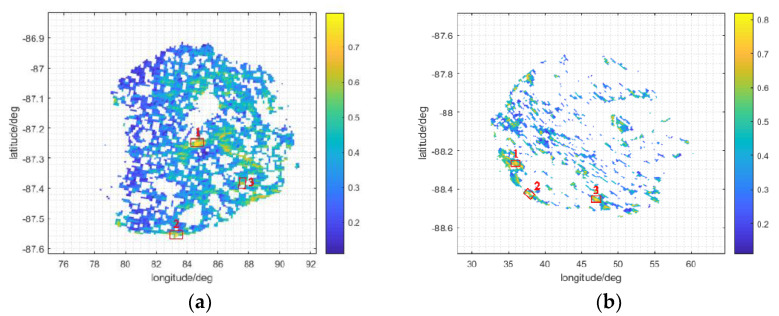
The fuzzy evaluation results and partially suitable areas marked with solid red boxes in the Faustini (**a**) and Shoemaker (**b**) craters.

For each crater, the three sites with relatively higher evaluation index F, expressed as the single output in Figure 6, are indicated in Figure 15 with red boxes. The evaluation index F is an absolute index in [0, 1]; the higher the index, the more suitable the site. The detailed statistics of each red box in Figure 15 are displayed in Table 1.

From Figure 15, we can see that the time coverage of Earth disc observation at suitable sites is also affected by topography once high-resolution data are used instead of coarse data, as shown in Figure 9. We also calculated the average F index value in Faustini and Shoemaker as 0.376 and 0.341, respectively. By comparing the average F values of the two craters, Faustini was found to be better than Shoemaker, but this does not mean a site cannot be selected in the Shoemaker crater; the evaluation index F values of some sites in Shoemaker are equal to those in Faustini. The most suitable sites are basically distributed in the edge regions, the inappropriate areas of which are all removed, because in this kind of edge region, the site’s slope meets its maximum constraint ($S_{max}$ in Equation (15)), and the topography is relatively flat. In Table 1, the maximum site slope of the labeled red boxes in Figure 15 is about 1.6°, far less than the slope constraint 5.71°. The surrounding area has a relatively high F, as shown in Figure 15, which indicates an area buffer of red boxes that satisfy the safety requirement of avoiding falling rocks. The occlusion of sunlight also meets the required conditions regarding light constraints shown in Equation (16).

To more specifically illustrate the distribution of suitable sites, Table 2 lists the percentages of F values in different intervals, showing that the period for which the *F* value is less than 0.1 and greater than 0.7 is small, and the remaining intervals are big, so there is no contiguous concatenation area that possesses F values higher than 0.7, but there are several sites that possess a high F, as shown by the red boxes in Figure 15.

### 3.3. Application of the Evaluation Model to Site Selection for Earth-Related Plasma/Magnetosphere Observation

**Figure 16:**
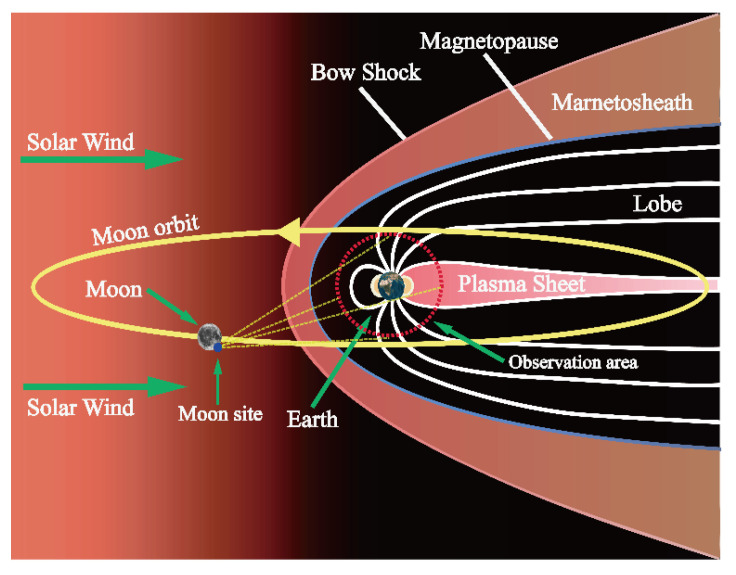
The observation of the plasmasphere/magnetosphere from a Moon site (the figure was created by Adobe Illustrator, vector graphics software).

A Moon-based Earth observation station should not only cover the whole Earth disc, but also an area that includes plasmasphere and magnetosphere information, as shown in Figure 16.

For other scientific research, the observation object of interest is the plasmasphere/magnetosphere, instead of the whole Earth disc. Most of the time, e.g., for about 75% of its orbital period, the Moon is in the path of solar wind and exposed to the direct impact of solar wind radiation, which is a stream of supersonic particles (mainly electrons and protons) ejected from the outer layer of the Sun’s atmosphere, and this continues to affect the distribution and intensity of the plasmasphere/magnetosphere. During the remaining time, about 25% of its orbital period, the Moon is in the path of the Earth’s magnetic field, and passes through the magneto-sheath, plasma sheet, bow shock, and lobe, as shown in Figure 16 [38]. The Earth’s plasma layer is located above the Earth’s ionosphere, extending to an annular plasma area about 3–6 times the size of the Earth’s radius; its spatial distribution overlaps with the Earth’s radiation belt and ring current area, and it is an important aspect of research on the Earth’s inner magnetosphere [39]. As such, conducting Earth observations from the Moon will contribute to research on regulating the dynamic characteristics of the Earth’s radiation belt, and to research about the Sun’s surface and internal activities. A Moon-based station located in the south pole area would have a good thermal environment, and the unevenly distributed craters would provide natural conditions for radiation protection.

As shown in Figure 16, any of the red boxes in Figure 15 can be selected as the Moon site in the Faustini and Shoemaker craters. Their observation areas are 3 and 5 times the Earth’s radius, respectively, and contain information on the plasmasphere/magnetosphere. The coverage of the object of interest of the red boxes in Figure 15 and the corresponding evaluation factor F are provided in Table 3.

From Table 1 and Table 3, we can determine that the time coverage of the object of interest decreases with increasing observation area. When the observation area is equivalent to the Earth’s radius, e.g., the object of interest is the whole Earth disc, the time coverages with and without DEM both exceed 0.4; when the observation area is 3 and 5 times the Earth’s radius, the time coverages with and without the DEM decrease to 0.3~0.4 and 0.2~0.3, respectively. Increasing the observation area increases the field of view and enables the boundary to reach the topographical restriction. Because the Moon sites’ DEM of the red boxes shown in Figure 15 for the Faustini and Shoemaker craters are below the standard without the DEM, the time coverage of the interested object without the DEM will be a little larger than the time coverage with the DEM. Through the MISO method, the renewed non-zero evaluation factor F shows that craters in the south pole area are suitable for use as the site of a Moon-based station, not only for whole-Earth disc observation, but also for observing the Earth’s plasmasphere/magnetosphere.

## 4. Conclusions

The lunar south pole region can be a candidate area for site selection of a Moon-based station. When the Moon station is used for Earth observation, e.g., whole-Earth disc observation or Earth-related plasmasphere/magnetosphere observation, the topography of the Moon’s surface should be considered during research on the slope, the time coverage of Earth observation, and the time coverage of sunlight. During actual site selection, the time coverage of sunlight determined without considering topography is markedly different from that determined with a consideration of topography; conversely, the time coverage of Earth observation both with and without topography consideration shows little difference when the coarse data are used, but still more details are obtained when higher-resolution data are used. During the period of the Moon’s orbit around the Earth, polar day and night occur at high latitudes in the south pole region, including at the Faustini and Shoemaker craters. The low DEM and moderate apparent depth indicate that some permanently shadowed areas exist in the bottoms of the craters, providing a better thermal environment and radiation protection. The Faustini and Shoemaker craters were selected as the two main research regions in our study, based on a MISO approach. The analysis results show that there are no contiguous concatenation areas with high evaluation indexes that are suitable for establishing a Moon base, but several suitable sites are distributed along the edges of inner-crater hills, potholes, and uplifts, the inappropriate areas of which were removed.

The simulation in this paper also showed that through the MISO method, we can select a site on the Moon’s surface, and ensure the selected sites have a buffer of surrounding areas to satisfy the safety need of avoiding falling rocks. We input three factors into the MISO in this study; more factors and complex fuzzy rules can also be applied in this MISO method in future, if more factors need to be considered.

## Figures and Tables

**Table 1:** The evaluation index F and other performance parameters of red boxes shown in Figure 15.

	Faustini			Shoemaker		
Box number	1	2	3	1	2	3
Latitude	87.285° S	87.551° S	87.383° S	88.292° S	88.431° S	88.451° S
Longitude	83.612° E	82.917° E	87.501° E	36.255° E	38.187° E	47.041° E
slope	0.563°	1.612°	0.778°	0.321°	0.738°	1.306°
Coverage of sunlight	0.106	0.119	0.121	0.104	0.113	0.098
Coverage of Earth disc with DEM	0.433	0.439	0.406	0.487	0.494	0.463
Coverage of Earth disc without DEM	0.454	0.454	0.445	0.505	0.498	0.489
Membership of slope	0.902	0.718	0.864	0.944	0.871	0.771
Membership of sun light coverage	0.936	0.810	0.791	0.963	0.875	0.983
Membership of Earth disc coverage	0.953	0.967	0.912	0.965	0.991	0.944
F	0.803	0.774	0.755	0.819	0.819	0.788

**Table 2:** The percentage of F in the different intervals shown in Figure 15.

F	Faustini	Shoemaker
(0, 0.1)	0%	0%
(0.1, 0.3)	28.72%	41.75%
(0.3, 0.5)	52.44%	45.02%
(0.5, 0.7)	17.68%	11.23%
(0.7, 0.9)	1.16%	2%
(0.9, 1)	0	0

**Table 3:** The coverage performance and corresponding F of the red boxes in Figure 15.

	Faustini			Shoemaker		
Box number	1	2	3	1	2	3
Coverage of observation area (3 times) with DEM	0.338	0.344	0.313	0.398	0.403	0.376
Coverage of observation area (3 times) without DEM	0.359	0.359	0.349	0.413	0.407	0.398
F (3 times)	0.793	0.768	0.744	0.817	0.819	0.788
F (3 times to 1 time)	0.664	0.662	0.649	0.680	0.689	0.671
Coverage of observation area (5 times) with DEM	0.222	0.230	0.196	0.301	0.302	0.279
Coverage of observation area (5 times) without DEM	0.247	0.247	0.234	0.314	0.305	0.295
F (5 times)	0.760	0.747	0.706	0.813	0.819	0.790
F (5 times to 1 time)	0.487	0.506	0.444	0.595	0.594	0.572

The term in brackets “3 times” indicates the corresponding result was calculated in an observation area about 3 times the size of the Earth’s radius. “3 times to 1 time” indicates the result with DEM was calculated in an observation area about 3 times the Earth’s radius, and the results for comparison without DEM were calculated in an observation area about the same as the Earth’s radius. For the results with/without DEM, we used Equation (17). The contents in other brackets are similar to those above.

## Appendix A

The fuzzy rules used in this study are provided below. When formulating the fuzzy rule table, factor $u_{3}$ is considered the main factor, the second factor is $u_{2}$, and the third is $u_{1}$, so the categories in these tables are symmetrical along the main diagonal.

When factor $u_{3}$ is IC_3_, the evaluation output table is as shown in Table A1.

**Table A1:** The fuzzy rule table when $u_{3}$ is IC_3_.

	HA_2_	IA_2_	LA_2_	IC_2_
HA_1_	LA	LA	IC	IC
IA_1_	LA	IC	IC	IC
LA_1_	IC	IC	IC	IC
IC_1_	IC	IC	IC	IC

When factor $u_{3}$ is LA_3_, the evaluation output table is as shown in Table A2.

**Table A2:** The fuzzy rule table when $u_{3}$ is LA_3_.

	HA_2_	IA_2_	LA_2_	IC_2_
HA_1_	LA	LA	LA	LA
IA_1_	LA	LA	LA	LA
LA_1_	LA	LA	IC	IC
IC_1_	LA	LA	IC	IC

When factor $u_{3}$ is IA_3_, the evaluation output table is as shown in Table A3.

**Table A3:** The fuzzy rule table when $u_{3}$ is IA_3_.

	HA_2_	IA_2_	LA_2_	IC_2_
HA_1_	IA	IA	IA	LA
IA_1_	IA	IA	LA	LA
LA_1_	IA	LA	LA	IC
IC_1_	LA	LA	IC	IC

When factor $u_{3}$ is HA_3_, the evaluation output table is as shown in Table A4.

**Table A4:** The fuzzy rule table when $u_{3}$ is HA_3_.

	HA_2_	IA_2_	LA_2_	IC_2_
HA_1_	HA	HA	IA	IA
IA_1_	HA	HA	IA	LA
LA_1_	IA	IA	LA	LA
IC_1_	IA	LA	LA	IC
